# Online group psychotherapy during the “lock down”

**DOI:** 10.1192/j.eurpsy.2021.2085

**Published:** 2021-08-13

**Authors:** D. Romac

**Affiliations:** Department Of Mental Health And Addiction Prevention, Teaching Institute for public health Dr Andrija Štampar, Zagreb, Croatia

**Keywords:** group, online psycotherapy, “lock down”

## Abstract

**Introduction:**

The Covid-19 pandemic has limited the classic psychotherapy treatment. EAP provided temporary recommendations for online psychotherapy in March of 2020 which brought new possibilities and limitations and contains rules, ethics and techniques. From the perspective of a psychodynamic therapist, the specificity of online group psychotherapy in the context of strong stressors is described.

**Objectives:**

During the “lockdown” period and the earthquakes that occurred simultaneously in Zagreb in March of 2020, the secure Internet platform enabled the continuity of the group’s work in a video link modality.

**Methods:**

Online group had regular weekly meetings which lasted 1.5 hours. The classic rules of group analytical therapy were adapted to the new setting in virtual space. A risk assessment was also performed.

**Results:**

The six group members and therapist have connected online through more freedom, developed new levels of sensitivity, flexibility and creativity. They have also accepted limitations. The online group is able to provide holding, but deeper connections require a physical presence to exchange emotions that technology interferes with as well as the process of empathizing. Previous live sessions crucial to maintaining emotional connections have served as reservoirs for a period in which communication over the Internet was insufficient.

**Conclusions:**

Internet technology can temporarily enable the continuity of a group psychotherapy. Technical and institutional support is recommended. The advantages of technology can be used if the technique is adapted, realistic goals set, and clinical limitations accepted. Many questions about the possibilities of “online psychotherapy” are open and unexplored.

**Disclosure:**

No significant relationships.

